# Importance of Soil Temperature for the Growth of Temperate Crops under a Tropical Climate and Functional Role of Soil Microbial Diversity

**DOI:** 10.1264/jsme2.ME17181

**Published:** 2018-04-28

**Authors:** Nurul Syazwani Ahmad Sabri, Zuriati Zakaria, Shaza Eva Mohamad, A Bakar Jaafar, Hirofumi Hara

**Affiliations:** 1 Department of Environmental Engineering and Green Technology, Malaysia-Japan International Institute of Technology, Universiti Teknologi Malaysia (UTM) Kuala Lumpur Malaysia; 2 Ocean Thermal Energy Centre, Universiti Teknologi Malaysia (UTM) Kuala Lumpur Malaysia; 3 Department of Chemical Process Engineering, Malaysia-Japan International Institute of Technology, Universiti Teknologi Malaysia (UTM) Kuala Lumpur Malaysia

**Keywords:** soil cooling, tropical environment, temperate crops, microbial diversity

## Abstract

A soil cooling system that prepares soil for temperate soil temperatures for the growth of temperate crops under a tropical climate is described herein. Temperate agriculture has been threatened by the negative impact of temperature increases caused by climate change. Soil temperature closely correlates with the growth of temperate crops, and affects plant processes and soil microbial diversity. The present study focuses on the effects of soil temperatures on lettuce growth and soil microbial diversity that maintains the growth of lettuce at low soil temperatures. A model temperate crop, loose leaf lettuce, was grown on eutrophic soil under soil cooling and a number of parameters, such as fresh weight, height, the number of leaves, and root length, were evaluated upon harvest. Under soil cooling, significant differences were observed in the average fresh weight (*P*<0.05) and positive development of the roots, shoots, and leaves of lettuce. *Janthinobacterium* (8.142%), *Rhodoplanes* (1.991%), *Arthrospira* (1.138%), *Flavobacterium* (0.857%), *Sphingomonas* (0.790%), *Mycoplana* (0.726%), and *Pseudomonas* (0.688%) were the dominant bacterial genera present in cooled soil. Key soil fungal communities, including *Pseudaleuria* (18.307%), *Phoma* (9.968%), *Eocronartium* (3.527%), *Trichosporon* (1.791%), and *Pyrenochaeta* (0.171%), were also recovered from cooled soil. The present results demonstrate that the growth of temperate crops is dependent on soil temperature, which subsequently affects the abundance and diversity of soil microbial communities that maintain the growth of temperate crops at low soil temperatures.

Climate has adversely changed over the past few decades, and is projected to continue increasing from 1.8°C to 4.0°C by the end of this century (30). Climate change is attributed to the increase in greenhouse gases, including carbon dioxide (CO_2_), methane (CH_4_), and nitrous oxide (N_2_O), resulting from human activities, such as the burning of fossil fuels for energy production, agricultural activities, deforestation, and global warming (12, 30, 64). Climate change markedly affects crop production biophysically by altering meteorological variables including temperature, precipitation, and atmospheric CO_2_ levels (49, 62). These biophysical effects of climate change vary with time and differ in some agricultural regions depending on the current climate, soil conditions, and crop variety (1, 48). The effects of climate change on food availability have been reported in temperate zones such as the European Union for wheat and the United States of America for maize (42). Both crops were shown to be negatively affected by reduced water availability during growing seasons, frequent heat events that damage the flowering stages, and accelerated phenology, which resulted in a decrease in biomass production (19, 42). Climate change is also expected to cause potential losses and damage to fruits and vegetables that are adapted to lower temperatures, such as grapes, apples, carrots, tomatoes, spinach, broccoli, lettuce, and other *Brassica* species (4, 32, 51).

Temperature increases attributed to climate change closely correlate with the development of temperate fruits and vegetables, and affect various plant processes including photosynthesis, respiration, water transport, membrane stability, plant hormones, and primary and secondary metabolites (38). Temperature changes also affect seed germination, leaf development, flowering, harvest, and fruit production (5, 41). However, the effects of temperature on crop development are often implicitly presumed to be associated with air, but not soil temperature. Soil temperature more closely correlates with crop development than air temperature (35, 54, 67, 70). Plant processes, including nutrient and water uptake, as well as the initiation, branching, and orientation of root growth are also dependent on soil temperature (33). A previous study conducted by González *et al.* demonstrated the effects of soil temperature (12°C vs. 25°C) on the growth and development of carrot cultivars. A soil temperature of 12°C resulted in a higher fibrous root (involved in the uptake of water and nutrients): shoot ratio, thicker and smaller leaves, and a higher taproot (stores carbohydrates and other compounds): shoot ratio (26). At low soil temperatures, berries showed increases in aromatic compounds, while tomatoes had higher sucrose contents in their leaves and greater maximum fruit production (68). Soil temperature also influences the susceptibility of crops to diseases. Previous studies demonstrated that the infection of sugar beet by *Polymyxa betae* was absent and delayed at soil temperatures of 10°C and 15°C, respectively (10), while the frequencies of various diseases, including brown stripe downy mildew (maize), charcoal rot (soybean), and cylindrocladium black rot (peanut), were higher in crops at high soil temperatures between 25°C and 37°C (45). These findings further justify the importance of low soil temperatures for the growth and development of high-quality temperate produce.

The growth of temperate crops is dependent on low soil temperatures, which also affect the abundance and diversity of soil microbial communities. Soil bacteria play diverse and significant roles in the nutrient cycling of all major elements, including carbon, nitrogen, and phosphorus, which subsequently affects the structure and function of soil maintaining the growth of temperate crops (3, 7, 27). Soil bacteria interact with each other and plants in various growth processes. Plant-microbial interactions may be negative when pathogenic, symbiotic mutualists and decomposers reduce plant performance and positive when the soil microbial community maintains plant growth and development (14). Beneficial microbes, such as *Bacillus*, *Pseudomonas*, *Microbacterium*, *Arthrobacter*, and *Lysobacter*, have been identified from potatoes and lettuce grown on temperate agricultural soils (34, 53).

In addition to these findings, we reported the efficiency of a soil cooling system that prepares soil for temperate soil temperatures for the growth of temperate crops under a tropical climate (submitted for publication). The model temperate crop, loose leaf lettuce, was grown on cooled and uncooled temperate eutrophic soil for a 3-growth cycle without the addition of fertilizer throughout the growth cycle. The average fresh weight and positive development of the root, shoot, and leaf for a 3-growth cycle was different for lettuce grown on cooled soil. In contrast, this study featured key soil microbial communities that maintain the growth of temperate crops, particularly during the 50-d/1^st^ growth cycle that showed significant differences in the lettuce fresh weight grown on cooled soil from other growth cycles, which also showed positive growth responses in contrast to uncooled soil. Few studies have investigated the relationship between soil microbial diversity and soil function in temperate agriculture. Therefore, the present study will focus on elucidating the functional role of soil microbial diversity that maintains the growth of temperate crops at low soil temperatures.

## Materials and Methods

### Field site Soil sampling

Eutrophic soil of the Cameron Highlands was collected from Mardi agricultural sites on 24^th^ March 2016 with geographical coordinates of 4.4679° N and 101.3847° E in order to study soil microbial diversity i) before and ii) after the growth of temperate crops under soil cooling. The physical and chemical properties of eutrophic soil are shown in Table S1. Soil from three sampling points was collected in order to assess soil bacterial diversity before crop growth using a soil probe at a depth of 20 cm from the soil surface. These soil samples were transported in a zip-lock bag and then stored in ice upon transferal to the laboratory. Soil samples were sieved using a 0.35-mm sieve to remove rocks and large organic debris or directly stored at 4°C until DNA extraction. In order to study soil bacterial and fungal diversities after the growth of temperate crops under soil cooling, approximately 300 kg of eutrophic soil was collected for the soil plot of the soil cooling system. Soil samples were kept in collection bags and transferred to the soil plot on ice on the same day of collection in order to preserve temperate soil conditions.

### Cooling of soil

We developed a soil cooling system to grow lettuce in a tropical climate (Fig. S1); a greenhouse consisted of a water chiller (DC-300; D-D The Aquarium Solutions), custom-made chilled water storage tank, exhaust fan, circulating and embedded pipes, and soil plots.

The soil plot with a dimension of 3×2×0.35 m comprised cooled and uncooled soil plots. Each cooled soil plot was installed with pipes embedded within the soil. The details of the system were as follows: the water chiller was set to 4°C to imitate the temperature of cold deep seawater and connected to the chilled water storage tank through circulating pipes. Tap water in the storage tank was cooled by cold water from the chiller. Chilled water was pumped from the storage tank to embedded pipes within the cooled soil plot in order to cool the soil. Water subsequently flowed back to the storage tank to be cooled by cold water continuously pumped out from the chiller and recirculated throughout the system. Soil was cooled by the constant circulation of chilled tap water throughout crop growth.

### Growth of temperate crops

The model temperate crop grown in the present study was loose leaf lettuce (*Lactuca sativa* L.). Lettuce seeds (633 Green Parade, Leckat) were sown indoors at a temperature between 21°C and 24°C until the sprouting of seeds was observed. Upon sprouting, seedlings were exposed to natural sunlight to prevent leggy seedlings for approximately 7 to 14 d or until the seedlings had more than four true leaves. The seedlings were then transplanted to cooled (*n*=20) and uncooled (*n*=20) soil plots at a spacing of approximately 15 cm between each lettuce. In the cooled soil plot, lettuce seedlings were transplanted next to the chilled pipes embedded within the soil (Fig. S2). Lettuce was harvested after a growing period of 50 d starting from the transplantation of lettuce seedlings to the harvesting of matured lettuce. Throughout lettuce growth, no additional fertilizers were added to the soil. Lettuce was watered every day in the morning and late evening at the top 6 inches of each watering session to ensure adequate moisture.

#### Measurement of air and soil temperatures

During lettuce growth, the soil temperature at each growing point was recorded using a soil thermometer at a depth of 20 cm, while air temperature inside the greenhouse was also recorded 10 cm from the soil surface using an air thermometer every hour for 24 h. Readings were averaged and shown as the mean±standard deviation (SD).

#### Sampling of the soil of harvested crops

Prior to the growth assessment, cooled and uncooled soil from representative matured lettuce was sampled using a sterilized hand shovel. The hand shovel was used to carefully lift mature lettuce and the surrounding soil without damaging the roots. Soil surrounding the lettuce roots was sampled and stored at 4°C until DNA extraction.

#### Evaluation of crop growth

Matured lettuce was further evaluated for attributes including fresh weight, height, the number of leaves, and root length from cooled and uncooled soil upon harvest. The fresh weight of lettuce (*n*=3) in triplicate readings was averaged and expressed as the mean±standard deviation (SD). Lettuce height was measured as the vertical distance between the soil surface and highest part of the lettuce (46). The number of leaves was recorded by counting all visible leaves, including the tips of new emerging leaves. Root length was measured using a measuring tape to the nearest centimeter (cm) in triplicate readings and averaged (44).

#### Statistical analysis

The significance of differences was calculated from independent samples by the *t*-test in SPSS Statistics software (Version 21), with *P*=0.05 being regarded as significant.

### Analysis of soil microbial communities

#### DNA extraction and amplicon sequencing

Genomic DNA was extracted from soil samples using the Ezup Column Soil DNA Purification kit (Bio Basic, New York, USA) following the manufacturer’s standard protocol. The concentration and integrity of extracted DNA products were assessed using Qubit 2.0 Fluorometer (Thermo Fisher Scientific, CA, USA) and gel electrophoresis, respectively, to ensure sufficient DNA quantity and quality for the polymerase chain reaction (PCR). Hypervariable region, V4 of bacterial 16S rRNA and fungal Internal Transcribed Spacer (ITS) genes were amplified using composite primers specific to each domain (13, 23, 69) (Table S2). The size of PCR products was verified using the Bioanalyzer DNA 1000 chip and purified using Agencourt AMPure XP (Beckman Coulter, USA). Sequencing adapters and dual-index barcodes were added to the PCR products for library preparation. Amplicon sequencing was conducted using the Illumina MiSeq platform (Illumina, San Diego, CA, USA) at BGI, Hong Kong, China.

#### Data analysis

Raw data were filtered prior to a bioinformatics analysis to obtain more accurate and reliable results by removing reads contaminated by adapters, ambiguous bases (Ns), reads with a low complexity of 10 consecutive similar bases, the truncation of sequence reads with less than 75% of the original length, and an average quality of 20 sliding windows of 30 bp. The overlapping sequences of paired end reads were generated using FLASH (Fast Length Adjustment of Short reads, v1.2.11) by allowing minimal overlap with a length of 15 bp, and a mismatch ratio ≤0.1 for the overlapped region. The high-quality paired-end reads for 16S sequencing were then combined to tags based on the overlap, generating 3,782,738 tags with an average length of 253 bp. Regarding ITS sequencing, the removal of paired end reads without an overlap was followed by the removal of primer sequences. Forward and reverse amplification primer sequences were retained from the reads if 4 consecutive bases at the 3′-end of the primer completely matched the tags with mismatch bases less than 2. Upon the removal of primers, paired end reads of ITS sequencing generated 936,942 tags with an average length of 238 bp. The tags were clustered into Operational Taxonomic Units (OTU) at 97% sequence similarity using USEARCH (v7.0.1090). 16S rDNA and ITS sequences were screened and filtered for chimera with UCHIME (v4.2.40) using gold database (v20110519) and UNITE (v20140703), respectively, as a reference template. All tags were mapped to each OTU representative sequence using USEARCH GLOBAL and summarized into OTU abundance. The taxonomic assignment of the representative OTU sequences was then classified using Naїve Bayesian Classifier (v.2.2) provided by the Ribosomal Database Project (RDP) and trained on the Greengene database at a 0.6 confidence threshold for bacterial 16S and 0.8 confidence threshold for fungal ITS. Species annotation for bacterial and fungal communities were assessed using Greengene (default) V201305 and UNITE (default) Version 6 20140910, respectively. Alpha (α)-diversity indices, including Chao1, ACE, and the Shannon diversity index, were calculated by Mothur v1.31.2 and the corresponding rarefaction curve was generated using software R v3.1.1. In order to evaluate differences within the bacteria and fungi assemblages between cooled and uncooled soil samples, a beta (β)-diversity analysis including Bray-Curtis and weighted and unweighted UniFrac was conducted using software QIIME (v1.80). Differences in communities between cooled and uncooled soil samples were then assessed graphically using the ordination method of multidimensional scaling (MDS) according to the matrix of the Bray-Curtis distance.

## Results

### Effects of soil temperature on the growth of temperate crops in a tropical climate

A greenhouse with a soil cooling system was used to grow loose leaf lettuce under tropical climate conditions. The air temperature of the greenhouse was 31.6±5.8°C during lettuce growth. Under soil cooling, the temperature of cooled soil was 16.5±2.1°C, while that of uncooled soil was 28.4±1.7°C.

Lettuce was grown on cooled and uncooled soil for 50 d, photographed (Fig. S3), and its fresh weight, height, number of leaves, and root length were evaluated upon maturation. Table 1 shows the fresh weight, height, root length, and number of leaves of lettuce grown on cooled and uncooled soil. Upon harvest, a significant difference was observed in the average fresh weight of lettuce grown on cooled (12.4±2.5 g) and uncooled soil (2.24±0.9 g) (*P*<0.05). A significant difference was observed in the height of lettuce grown on cooled (16.2±0.4 cm) and uncooled soil (12.0±2.0 cm) (*P*<0.05). Similarly, a significant difference was noted in the root length of lettuce grown on cooled (8.8±0.3 cm) and uncooled soil (7.3±0.1 cm) (*P*<0.05). Lettuce had a higher number of leaves when grown on cooled soil than on uncooled soil.

### Effects of soil temperature on soil bacterial and fungal diversities during the growth of temperate crops under a tropical climate

Following the quality filtering process and removal of chimeric sequences, soil bacterial communities reported 686,149 reads from eutrophic soil before lettuce growth, 833,656 reads from cooled soil, and 773,407 reads from uncooled soil, while fungal species showed 259,668 reads from eutrophic soil, 268,100 reads from cooled soil, and 255,924 reads from uncooled soil with 50 d of growth. At 97% similarity, the numbers of OTUs for soil bacterial communities recovered from eutrophic soil before lettuce growth, uncooled soil, and cooled soil were 1,146,980, and 1,117, respectively. Regarding fungal communities, OTU numbers from eutrophic, uncooled, and cooled soil were 128, 136, and 154, respectively.

**Fig. 1:**
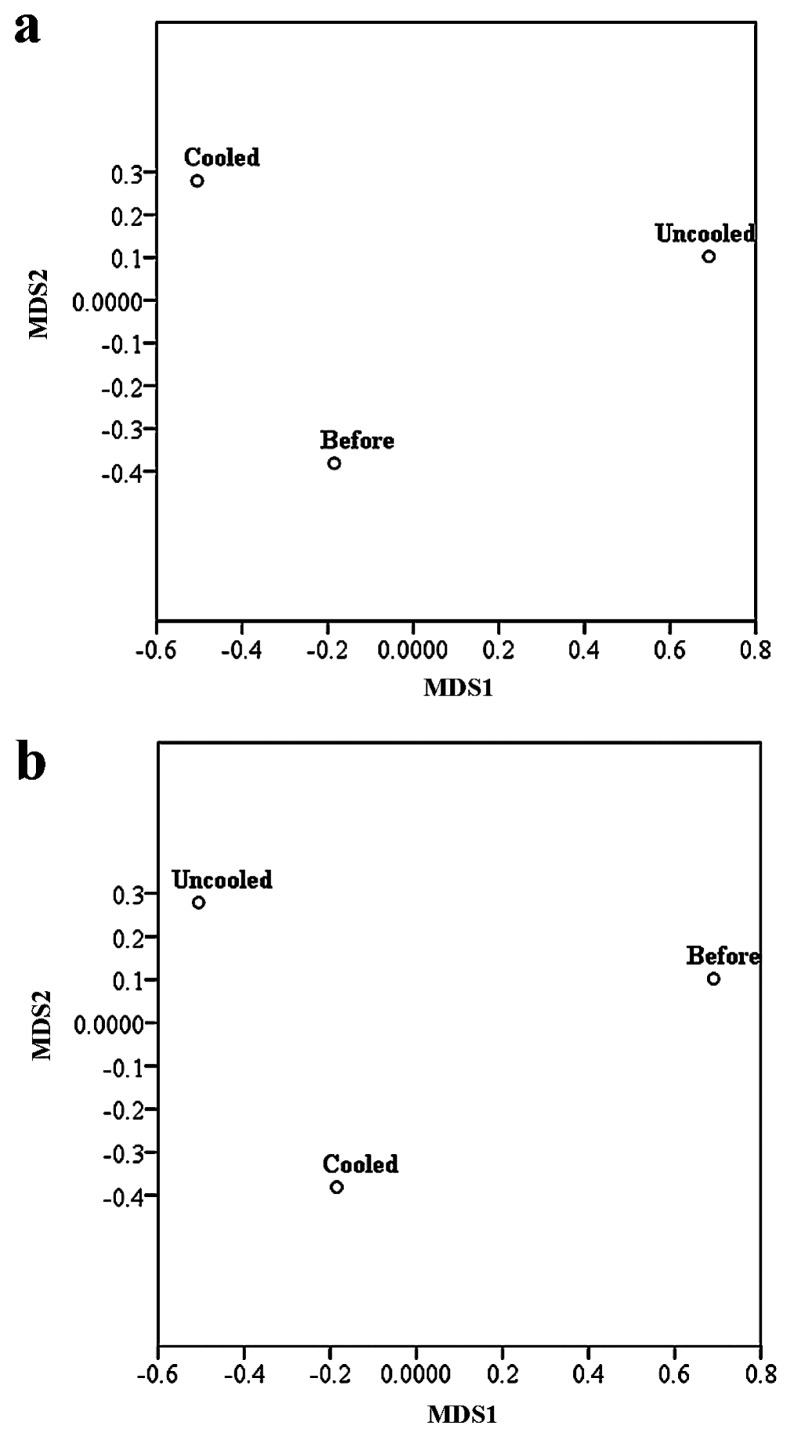
Multidimensional scaling (MDS) of overall bacterial (a) and fungal (b) communities between cooled and uncooled soil before and after lettuce growth according to the matrix of the Bray-Curtis distance. A closer distance indicates the similar species compositions of the samples.

Bacterial diversity decreased with the distance from eutrophic soil (before lettuce growth) to uncooled soil, while fungal diversity increased from eutrophic soil to cooled soil upon lettuce harvest (Fig. S4). Regarding bacteria, uncooled soil was reported to have the lowest number of observed OTUs, species richness Chao1 and ACE, and Shannon diversity index. In contrast, eutrophic soil scored the lowest value for Chao1 and ACE, whereas uncooled soil had the lowest value for the Shannon diversity index for fungal communities. Dissimilarities between bacterial and fungal diversities were shown in Fig. 1, and demonstrated the clear separation of community species between cooled and uncooled soil based on the matrix of the Bray-Curtis distance. In addition to Bray-Curtis, beta (β)-diversity values, including weighted and unweighted UniFrac, were also reported and summarized in Table S3. Regarding bacteria, the distance between eutrophic and cooled soil showed the highest values in Bray-Curtis, and weighted and unweighted UniFrac distances as compared to eutrophic and uncooled soils. Similar results were obtained for the Bray-Curtis and unweighted UniFrac distances of fungal communities, whereas a lower value was noted for weighted UniFrac.

In order to identify the key soil bacterial communities that maintain the growth of temperate crops at low soil temperatures, the relative abundance of bacterial genera in soil before and after lettuce growth was summarized in Table 2. Six main genera, including *Arthrospira* (1.138%), *Flavobacterium* (0.857%), *Janthinobacterium* (8.142%), *Mycoplana* (0.726%), *Pseudomona*s (0.688%), *Rhodoplanes* (1.991%), and *Sphingomonas* (0.790%), were more dominant in cooled soil than in uncooled soil. Similarly, soil fungal communities were recovered in eutrophic, uncooled, and cooled soil after 50 d of growth (Table 3). *Eocronartium* (3.527%), *Phoma* (9.968%), *Pseudaleuria* (18.307%), *Pyrenochaeta* (0.171%), and *Trichosporon* (1.791%) were the dominant genera recovered from cooled soil.

## Discussion

The soil cooling method using the principle of Ocean Thermal Energy Conversion (OTEC) serves as a potential platform for growing temperate crops under a tropical climate. OTEC utilizes temperature differences between warm surface seawater and cold deep seawater to generate electricity (21, 31). Besides producing electricity, the OTEC system may be used in the production of fresh water, discrete cooling for refrigerators and buildings, aquaculture, and temperate agriculture. The use of cold deep seawater for temperate agriculture has been implemented in Kume Island of Okinawa Prefecture and Hawaii. In Kume Island, cold deep seawater is pumped through an array of pipes that are embedded within soil. Cold deep seawater cools the surrounding soil and ensures the survival of crops such as spinach (Martin, B. 2017. Kume Guide: Agriculture. Available from: http://kumeguide.com/Industry/DeepSeaWater/ResearchInstitute/). In addition, cold deep seawater is utilized for the irrigation of grapes grown in the state of Hawaii (Yu, L. 2015. Natural energy lab fosters its own start up paradise. Hawaii Business Magazine. Available from: http://www.hawaiibusiness.com/natural-energy-lab-fostersits-own-startup-paradise/).

The potential of cold deep seawater to grow spinach and grapes under a tropical climate has led to the development of soil cooling systems. In order to study the effects of soil temperature on the growth of temperate crops under a tropical climate of 31.6±5.8°C, soil from agricultural plots in the Cameron Highlands was collected for the soil cooling system. The Cameron Highlands are one of the tropical regions in Malaysia that resemble a temperate climate, which is conducive for the growth of temperate produce. The soil of the Cameron Highlands is eutrophic and rich in organic nutrients due to the previous agricultural activities of temperate vegetables, including tomatoes, cabbage, and green mustard. Loose leaf lettuce was grown on eutrophic soil under soil cooling as a model temperate crop in the present study. Loose leaf lettuce was selected as a model temperate crop due to its high productivity in a limited space, easy germination indoors or when sown directly on soil, and short maturation time between 45 to 55 d (20, 61). Using this method, soil was cooled to 16.5±2.1°C, which is conducive for the growth of temperate crop varieties, particularly lettuce.

Ogbodo *et al.* previously reported that lettuce grew optimally at a temperature between 15 and 20°C (46). This finding explained the significant difference observed in the average fresh weight of lettuce grown on cooled soil. On uncooled soil, which had a soil temperature of 28.4±1.7°C, lettuce had a significantly lower average fresh weight due to the high soil temperature. The adverse effects of soil temperature on lettuce growth are supported by Dimsey and Ogbodo *et al.*, who reported the stunted growth and low yield of lettuce at high soil temperatures (16, 46). Furthermore, soil temperature was demonstrated to affect leaf, root, and shoot phenologies during the 50-d growth period. Upon harvest, lettuce grown on uncooled soil was significantly smaller and had shorter root lengths as than that grown under soil cooling. These differences were speculated to be due to the effects of soil cooling on soil bacterial and fungal communities, which play significant roles during lettuce growth.

Soil bacterial and fungal communities are key determinants of soil nutrients, crop health, and overall crop productivity, and these microorganisms decompose organic matter, improve plant nutrients, and suppress pathogens and plant diseases (9). In the present study, the most dominant soil bacterial and fungal genera were identified in order to obtain a clearer understanding of the functional role of each microorganism interacting within these communities and with lettuce at low soil temperatures. The results obtained showed the abundance of six bacterial genera that maintain the growth of lettuce on cooled soil in the following order: *Janthinobacterium*> *Rhodoplanes*>*Arthrospira*>*Flavobacterium*>*Sphingomonas*> *Mycoplana*>*Pseudomonas*. *Janthinobacterium*, a member of *Burkholderiales*, is commonly isolated from temperate climates and grows optimally at a temperature of 4°C (24). *Janthinobacterium* has functional genes that are related to nitrogen and phosphorus, including urea transport, dissimilatory nitrate reduction, denitrification, and phosphonate transport, which are vital for soil function (15, 18, 56). Some of the members of *Janthinobacterium* also contain genes that allow survival upon nutrient starvation and osmotic stress, and produce anti-microbial compounds that influence predator-prey and microbial-host symbioses (6, 39, 56).

Besides the most dominant *Janthinobacterium*, plant growth-promoting bacteria (PGPR), including *Sphingomonas*, *Pseudomonas*, and *Rhodoplanes*, were also recovered from cooled soil. These microorganisms were previously reported to be dominant in the organic farming system and capable of fixing atmospheric nitrogen, solubilizing phosphorus, and enhancing the production of plant hormones (55, 57). *Sphingomonas* and *Pseudomonas*, both produce the phytohormone auxin (indole-3-acetic acid/indole acetic acid/IAA), which is involved in every aspect of plant growth and development by affecting plant cell division, extension and differentiation, photosynthesis, and pigment formation, as well as the biosynthesis of various metabolites and resistance against environmental stress (2). IAA also facilitate increases in the root surface and length, which provides greater access for plant-to-soil nutrients, and loosening of plant cell walls, thereby increasing the amount of root exudates to support the growth of rhizosphere bacteria (25). Besides producing phytohormones, *Pseudomonas* is also involved in phosphorus cycling through phosphate solubilization and acts as a biocontrol agent that suppresses plant diseases by protecting seeds and roots from fungal infections (52, 60). Regarding *Rhodoplanes*, these microorganisms have the ability to fix atmospheric nitrogen along with cyanobacteria *Arthrospira* and several strains of diazotrophic *Mycoplana* (47, 50, 58, 59, 66). *Flavobacterium*, which accounts for approximately 30% of all bacterial genera in the rhizosphere of lettuce, is also involved in the production of the phytohormone auxin, the mineralization of various organic matters, and the solubilization of insoluble phosphate (mineral phosphate) compounds (8, 52, 65).

Various soil fungal genera were also recovered from cooled soil in the following order: *Pseudaleuria*>*Phoma*>*Eocronartium*> *Trichosporon*>*Pyrenochaeta*. *Pseudaleuria*, a member of the phylum Ascomycota, was mostly abundant in healthy soils (71). These fungal species are nutritionally saprobic on soil and/or wood, or form ectomycorrhizal symbioses with host plants (37, 43). Similarly, members of *Trichosporon*, including *Trichosporon dulcitum*, *T. laibachii*, *T. moniliiforme*, and *T. porosum*, which are common in the soil of temperate climates, are also saprobes. This genus has the ability to utilize polysaccharides that comprise plant constituents (*e.g.* xylan and galactomannan) and products excreted by bacteria (*e.g.* dextran) as carbon and energy sources (40). In contrast to *Trichosporon*, saprophytic *Pyrenochaeta* was ubiquitously found in the soil, plants, and woods of tropical and subtropical areas (36). Nevertheless, this study demonstrated the presence of *Pyrenochaeta* in cooled soil, indicating the role of this genus in maintaining the growth of lettuce. The genus *Pyrenochaeta* is more poorly defined than other fungal genera; however, previous studies demonstrated the ability of this genus to decompose polysaccharides (*e.g.* cellulose, hemicellulose, pectin, and xylan) as carbon sources (17, 28, 63). Similar to *Pyrenochaeta*, the PGPF *Phoma* was also shown to be capable of degrading cellulose (17). This PGPF has been reported to produce abscisic acid, which promotes plant growth, increases the production of ammonium, suppresses plant pathogens, and induces systemic resistance (29). In contrast to the aforementioned fungal genera that maintain lettuce growth, *Eocronartium*, which establishes a unique biotrophic relationship with temperate moss genera, was shown to dominate in cooled soil. This genus was restricted to bryophytes, which exploit nutrients that pass through the transfer cells of moss (11, 22). The abundance of *Eocronartium* in cooled soil is speculated to be due to the growth of mosses that co-exist with the lettuce grown in the present study. However, the growth of lettuce was shown to be unaffected by the presence of these mosses due to the abundance of *Eocronartium*, which subsequently parasitize mosses, thereby preventing nutrient competition between mosses and lettuce grown on cooled soil. Therefore, we herein identified and characterized a group of soil bacterial and fungal communities that play significant roles in regulating the nutrients of eutrophic soils without any additional treatments with organic and chemical fertilizers throughout lettuce growth.

This is a novel study that demonstrated the importance of soil cooling for growing temperate crops in a tropical climate, and identified the key soil bacterial and fungal communities that contribute to lettuce growth, which is only possible at temperate soil temperatures. Due to the importance of soil temperatures for temperate crop production in a tropical climate, this soil cooling system may also be used to grow other temperate crop varieties, including root crops, such as carrots, radish, potato, and beet. The use of soil cooling for temperate agriculture under a tropical climate allows the production of temperate crops throughout the year. However, the limitation of the present study is that the current soil cooling system was only able to grow crops on a small-scale. In addition, fertilizers and other agricultural practices are needed in order to produce large-scale marketable crops; however, this was not the main focus of the present study. In addition, gaps in current knowledge on the soil bacterial and fungal diversities of temperate agricultural soil also make it difficult to understand the functions of each microorganism during crop growth. Hence, further studies are needed in order to identify the active functional genes of each soil microbial community during crop growth under low and high soil temperatures.

## Supplementary Material



## Figures and Tables

**Table 1:** Fresh weight, height, number of leaves, and root length of lettuce grown on cooled and uncooled soil.

Soil cooling	Fresh weight (g)	Height (cm)	Number of leaves	Length of roots (cm)
Uncooled	2.24±0.9	12.0±2.0	7	7.3±0.1
Cooled	12.4±2.5*	16.2±0.4*	12	8.8±0.3*

* *P*<0.05 significantly different from uncooled soil.

**Table 2:** Relative abundance of soil bacterial genera recovered in soil before and after the growth of lettuce under soil cooling. Only bacterial reads that >1% for either one of the soil groups are represented in the table.

Genus	Before growth (%)	After growth (%)	

		Uncooled	Cooled
*Adhaeribacter*	0.148	1.867	1.201
*Arthrobacter*	4.96	4.101	1.995
*Arthrospira*	0	0.083	1.138
*Flavisolibacter*	0.043	5.229	1.753
*Flavobacterium*	0.029	0.048	0.857
*Janthinobacterium*	0.855	6.078	8.142
*Kaistobacter*	11.605	14.232	9.241
*Luteimonas*	0.311	1.207	0.679
*Lysobacter*	0.693	10.765	3.061
*Mycoplana*	0.005	0.367	0.726
*Perlucidibaca*	0	0.004	0.004
*Polaromonas*	1.122	0.625	0.323
*Pseudomonas*	0.081	0.022	0.688
*Rhodococcus*	0.301	2.440	0.267
*Rhodoplanes*	3.994	1.806	1.991
*Sphingomonas*	1.261	0.700	0.790
*Thermomonas*	0.148	1.570	1.286

**Table 3:** Relative abundance of soil fungal genera recovered in soil before and after lettuce growth under soil cooling. Only fungal reads that >1% for either one of the soil groups are represented in the table.

Genus	Before growth (%)	After growth (%)	

		Uncooled	Cooled
*Alternaria*	0.180	1.60	0.431
*Cladosporium*	0.868	2.829	0.250
*Eocronartium*	0	0.190	3.527
*Mortierella*	30.574	54.916	37.081
*Phoma*	2.329	0.379	9.968
*Pseudaleuria*	0.033	0	18.307
*Pyrenochaeta*	3.529	0.118	0.171
*Trichosporon*	0.043	0.071	1.791
