# Supplementary material for: Importance of Soil Temperature for the Growth of Temperate Crops under a Tropical Climate and Functional Role of Soil Microbial Diversity
Source: Microbes Environ. 2018 Apr 28;33(2):144–50. doi: 10.1264/jsme2.ME17181 (PMC6031386; doi:10.1264/jsme2.ME17181)
Supplement: Supplementary file 1 [file 33_144_s1.pdf]

Supplementary materials

Fig. S1. Soil cooling system established in 'OTEC' greenhouse. The 'OTEC' greenhouse consist of water chiller, circulating and embedding pipes, custom-made chilled water storage tank, exhaust fan, and soil plots for cooled and uncooled soils. A cooled soil plot was installed with embedded pipes connected to chilled water storage tank to cool the soils. Tap water inside the storage tank was kept cool with chilled water that is continuously pumped out from the chiller and recirculates throughout the system.

Fig. S2. Transplantation of lettuce seedlings to cooled soil plot. Lettuces were transplanted next to chilled pipes embedded within the cooled soil plots. Chilled water was continuously pumped through the embedding pipes to cool the surrounding soils, providing conducive conditions for lettuce growth.

Fig. S3. Representative of lettuces grown on cooled and uncooled soils photographed upon harvest.

Fig. S4. Rarefaction curves for bacterial (a) and fungal (b) communities before and after the lettuce growth. α- Diversity indices are listed.

Table S1. Physical and chemical properties of eutrophic soils. N/A refers to not available.

Table S2. Primers used in this study.

Table S3. Beta (β)-diversity values to evaluate the differences of bacterial and fungal communities between cooled and uncooled soils after the growth of lettuce.

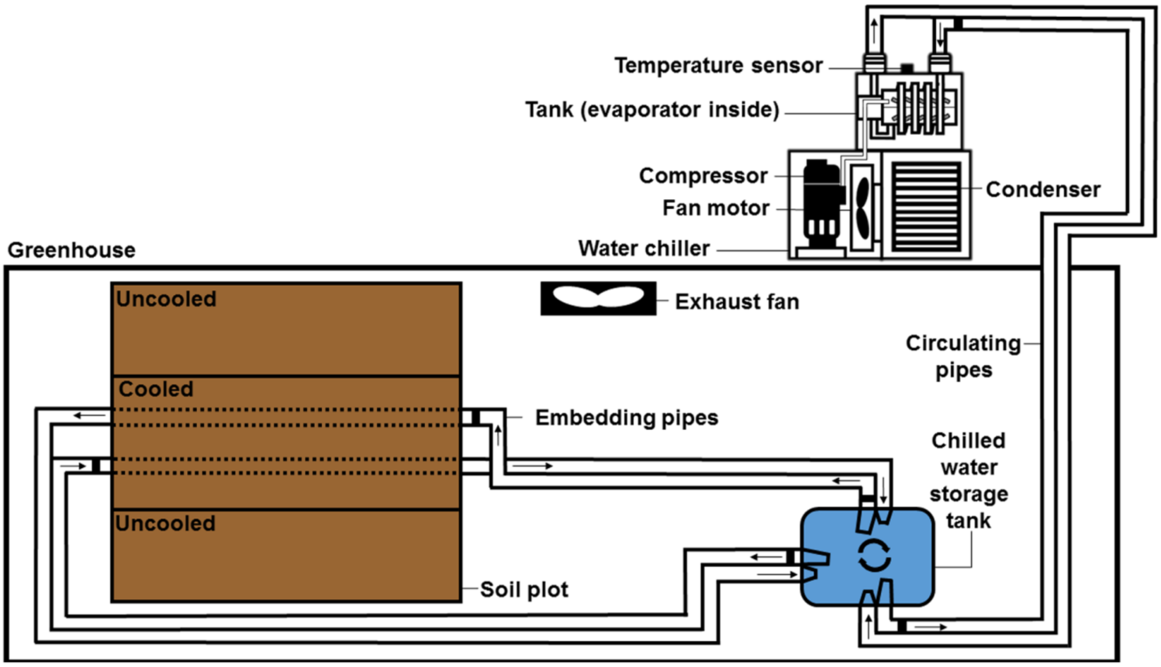

Fig. S1. *Nurul Syazwani et al.,*

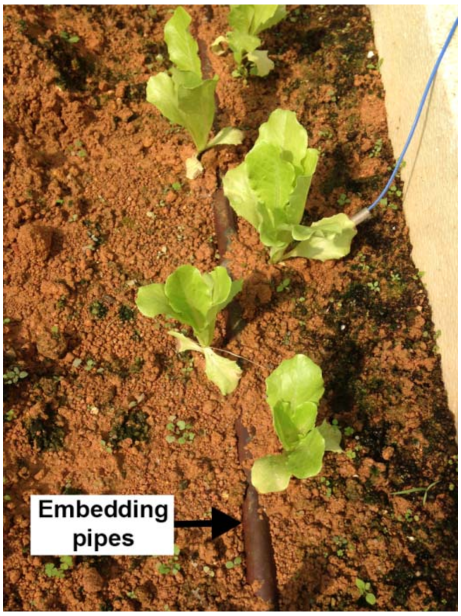

Fig. S2. *Nurul Syazwani et al.,*

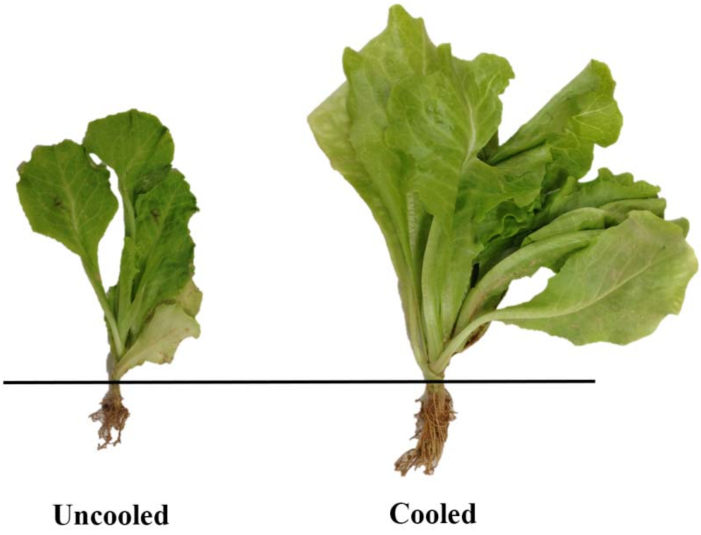

Fig. S3. *Nurul Syazwani et al.,*

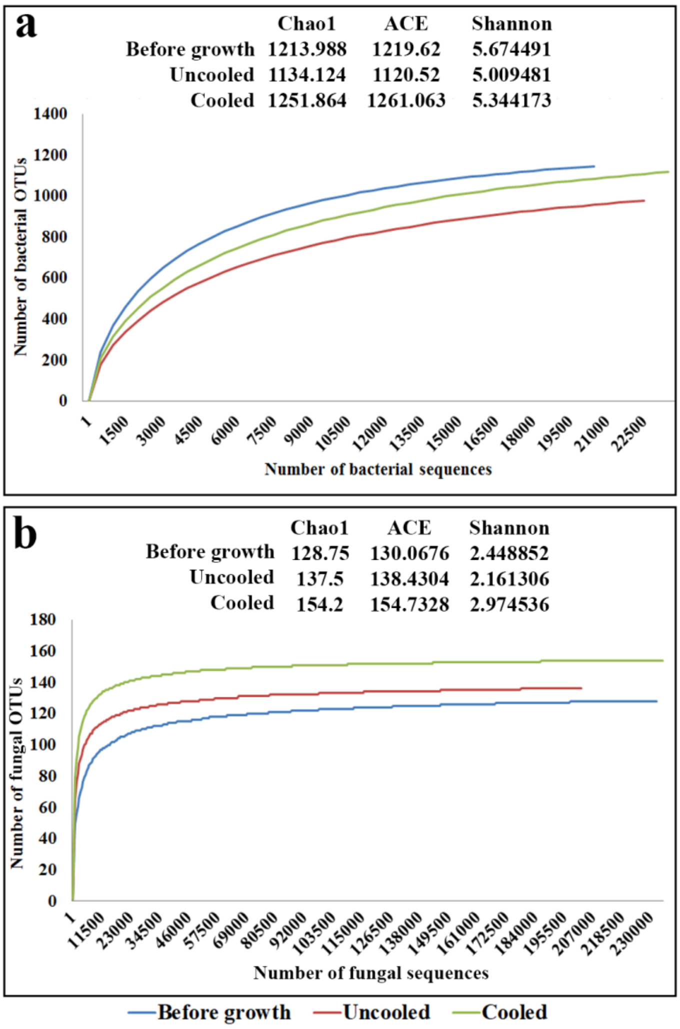

Fig. S4. *Nurul Syazwani et al.,*

Table S1. Physical and chemical properties of eutrophic soils. N/A refers to not available.

**Physical properties**	
Sand (%)	89.1
Silt (%)	10.5
Clay (%)	0.4
Textural Class	Loamy Sand
**Chemical properties**	
pH	6.66
Organic matter (%)	22.70
Nitrogen (%)	45.71
Phosphorus (%)	0.47
Potassium (mg/kg)	6.11
Calcium (mg/kg)	N/A
Magnesium (mg/kg)	176.70

Table S2. Primers used in this study.

Primer	Sequence (5'-3')"	Reference	Targeted region
**515F**	GTGCCAGCMGCCGCGGTAA	Caporaso et al.	Bacterial V4
**806R**	GGACTACHVGGGTWTCTAAT	Caporaso et al.	
**ITS-F**	TTGGTCATTTAGAGGAAGTAA	Gardes and Bruns	Fungal ITS1
**ITS-R**	GCTGCGTTCTTCATCGATGC	White et al.	

References

Caporaso, J.G., C.L. Lauber, W.A. Walters, D. Berg-Lyons, C.A. Lozupone, P.J. Turnbaugh, et al. 2011. Global patterns of 16S rRNA diversity at a depth of millions of sequences per sample. Proc Natl Acad Sci. 108: 4516–4522.

Gardes, M., and T.D. Bruns. 1993. ITS primers with enhanced specificity for basidiomycetes□application to the identification of mycorrhizae and rusts. Mol Ecol. 2: 113–118.

White, T.J., T. Bruns, S. Lee, and J.W. Taylor. 1990. Amplification and direct sequencing of fungal ribosomal RNA genes for phylogenetics. PCR Protocol: A Guide to Methods and Applications. 18: 315–322.

Table S3. Beta (β)-diversity values to evaluate the differences of bacterial and fungal communities between cooled and uncooled soils after the growth of lettuce.

		BACTERIA			FUNGI		
		Before	Uncooled	Cooled	Before	Uncooled	Cooled
**Bray-Curtis**	Before	0			0		
	Uncooled	0.467	0		0.553	0	
	Cooled	0.500	0.353	0	0.679	0.707	0
**Weighted UniFrac**	Before	0			0		
	Uncooled	0.230	0		1.029	0	
	Cooled	0.265	0.144	0	0.952	0.919	0
**Unweighted UniFrac**	Before	0			0		
	Uncooled	0.319	0		0.701	0	
	Cooled	0.329	0.311	0	0.716	0.748	0
